# Hexagonal Mesoporous Silica as a Rapid, Efficient and Versatile Tool for MALDI-TOF MS Sample Preparation in Clinical Peptidomics Analysis: A Pilot Study

**DOI:** 10.3390/molecules24122311

**Published:** 2019-06-22

**Authors:** Rosa Terracciano, Mariaimmacolata Preianò, Giuseppina Maggisano, Corrado Pelaia, Rocco Savino

**Affiliations:** 1 Department of Health Sciences, University “Magna Græcia”, 88100 Catanzaro, Italy; preiano@unicz.it (M.P.); giusimaggisano@unicz.it (G.M.); savino@unicz.it (R.S.); 2 Department of Medical and Surgical Sciences, University “Magna Græcia”, 88100 Catanzaro, Italy; pelaia.corrado@gmail.com

**Keywords:** MALDI-TOF, peptidomics, profiling, fingerprinting, biomarkers, sample preparation, analytical chemistry

## Abstract

Improvement in high-throughput MALDI-TOF MS analysis requires practical and efficient sample preparation protocols for high acquisition rates. The use of hexagonal mesoporous silica (HMS) sorbents in combination with MALDI-TOF MS was explored as a versatile tool for peptidomic profiling of clinical specimens difficult to process, but considered important sources of disease biomarkers: synovial fluid and sputum. A rapid and efficient procedure, based on dispersive solid-phase extraction of peptides using commercially available wormhole mesostructured HMS, was tested for: a) pre-concentration of standard peptides in serially diluted solution up to the sub-nanomolar range; b) peptidome profiling of sputum and synovial fluid. The use of HMS, as dispersed sponges, significantly amplified the peptidic repertoire of sputum and synovial fluid by excluding from the adsorptive process large size proteins, which mask and/or suppress peptidome signals. The protocol proposed, as dispersive solid phase extraction, ensures good analytical performances. Moreover, it is economical and rapid, as it avoids the use of less reproducible and prolonged sample preparation procedures, such as the use of ultrafiltration filter devices. These findings may contribute to defining a high-throughput screening MS-based platform for monitoring key peptidic features of difficult to analyse bodily fluids in a clinical setting.

## 1. Introduction

Peptidomics is a promising ‘‘omics’’ approach for the study and the qualitative–quantitative analysis of endogenous peptides in biological samples. The peptidome comprises not only naturally occurring peptides, including hormones, cytokines, and neurotransmitters, but also protein breakdown products originated from the action of active proteases. Stemming from the rationale that the activity of several proteases is regulated in different disease states, a difference in the degradation pattern of the target proteins is thus generated. Consequently, the resulting pattern of peptides discriminates between normal and pathological states and it can be useful to uncover details in the pathological mechanisms, thereby suggesting potential therapeutic interventions. The study of these peptides in biological matrices such as bodily fluids is, therefore, more significant than ever in clinical peptidomics [1]. However, the complexity and the high dynamic range of biological samples makes peptidomics even more challenging than proteomics [2]. In general, peptides are expressed in quite low abundance; moreover, they are frequently subjected to various post-translational processing, among which site-specific proteolysis by proteases and also proteasome degradation, making their detection challenging [3]. Abundant protein components (e.g., albumin in serum or plasma; mucins in induced sputum, hyaluronic acid polymers in synovial fluid) typically interfere with peptidomic analysis. Additionally, the presence in biological fluid of lipids, carbohydrates and salts gives rise to suppression effects, reducing the ionization efficiency of peptides [2]. Therefore, sample pretreatment is necessary prior to MS analysis for peptidomics-based investigations. Consequently, system-wide analysis of the peptidome continuously requires the development and improvement of novel protocols in order to detect low abundance peptides [1].

Matrix-assisted laser desorption/ionization time-of-flight (MALDI-TOF) mass spectrometry (MS) has recently captured significant attention in clinical chemistry for the ability of screening with high-throughput small amounts of clinical samples and for rapidly detecting biomolecules serving as disease biomarkers [4,5,6]. Comparative MALDI-TOF MS analysis may help in monitoring changes of expression patterns of disease specific peptides (between healthy and diseased individuals), with the potential to deliver fast and sensitive clinical assays. However, improvement in high-throughput MALDI-TOF MS analysis, especially in clinical applications, requires practical and efficient protocols for sample preparation in a form suitable for high acquisition rates.

Currently, a solid-phase extraction (SPE) step followed by MALDI-TOF MS represents a rapid and convenient tool for MS peptidomics profiling of clinical specimens [4]. Enrichment of the peptidome from clinical samples prior MALDI-TOF MS analysis is pursued by different procedures. In concomitance to well-known magnetic beads [7,8], or conventional SPE devices [9], innovative platforms and mesoporous materials for selective capture of peptides, phosphopeptides and proteins from bodily fluids and tissues, have been recently proved to be very successful [10,11,12]. In particular, given their highly ordered mesostructures, the large surface area of the pores, which accounts for up to 95% of the total surface of the material, mesoporous materials can directly interact and selectively capture biologically significant subproteomes from complex clinical matrices [13]. The most commonly used mesoporous materials for pre-treatment in proteomic analysis are those based on silica, owing to the facile synthesis and modifiable surface properties that turn advantageous for reducing non-specific adsorption or modulating selective adsorption during the enrichment step [10,11,13]. Indeed, mesoporous silica has captured considerable interest for a diverse range of applications, such as catalysis, filtration and separation, molecular collection and storage, nanofluidics, medical imaging, drug delivery, and sensors [14,15]. The porous structure properties of these silica sorbents, characterized by high pore volume and surface area, together with the pore size distribution were satisfactory for integrating size selectivity with adsorptive mechanism. In particular, the small pores and the surface properties provided the suitable prerequisites for developing an approach aimed to separate high-molecular weight from low-molecular weight proteins in biological clinical samples, such as bodily fluids. The uniform mesopores allow small-sized peptides access into mesoporous channels, while large-size proteins are excluded. In addition, the large surface area of mesoporous materials could provide several binding sites for modification; this improves the selectivity of materials and increases the sensitivity during MS analysis, thereby providing a suitable alternative to more traditional sorbents [6]. In our previous studies, we have reported that mesoporous silica with hexagonal pore arrangement (MCM-41 and SBA-15) can be used for the selective binding and enrichment of low molecular weight peptides of several bodily fluids [16,17,18]. In this work, we assayed the more stable mesostructured wormhole HMS silica with thicker pore walls, characterized by a pore size distribution in the range from 2 to 4 nm with a high pore volume 1–2 cm^3^/g and high surface area (800–1000 m^2^/g). In HMS, mesostructures typically form spongelike particles through the intergrowth of mesoscopic wormhole framework domains [19,20]. Compared to SBA-15, the smaller particle size of these sorbents, and the presence of wormhole framework domains facilitate the entrance in the pores and the entrapping of the analytes [21,22]. Recently, HMS were also used as new sorbent materials in food sample preparation [23]. As a part of an ongoing project aimed to screen the ability of mesoporous materials for selective isolation of peptides from clinical bio-fluids prior to MS analysis, wormhole mesostructured (hexagonal mesoporous silica) HMS, previously explored by our group for nasal fluid peptidome enrichment [16], were further investigated in this technical study for synovial fluid (SF) and spontaneous expectorate (sputum).

With the purpose of extensively assessing the array of applications in clinical peptidomics analysis, in the present investigation HMS were initially tested as harvesting sorbents for the analysis of diluted peptide mixtures. Then, as a proof of principle, pre-concentration and peptide enrichment of complex biological matrix such as one sputum and one SF sample were explored. A smart procedure was developed with the aim to provide a high-throughput MALDI-TOF MS-based platform for monitoring key peptidic-patterns useful for clinical biomarker investigations, after validation of its robustness by analysing several replicates of sputum and SF, subject of future work. 

## 2. Materials and Methods

### 2.1. Reagents

HMS (code 541036 wormhole silica mesostructured) was purchased from Sigma-Aldrich (St. Louis, MO, USA). Except when otherwise noted, all the chemicals used were analytical grade. Trypsin (proteomics grade), acetonitrile (ACN), water and trifluoroacetic acid (TFA) were purchased from Sigma Aldrich. Ammonium Bicarbonate and MALDI matrix alpha-cyano-4-hydroxy-*trans*-cinnamic acid (CHCA) were obtained from Fluka (St. Louis, MO, USA). ZipTip^®^C18 was purchased from Millipore Corporation (Billerica, MA, USA). Peptide standards were prepared from a peptide mass standards kit for calibration of AB SCIEX MALDI-TOF^TM^ instruments (AB Sciex, Framingham, MA, USA).

### 2.2. Preparation of Serial Diluted Solutions and Preconcentration Experiments with HMS

Peptide mass standards kit calibration mixture 1 (AB Sciex, Framingham, MA, USA) containing des-Arg^1^-bradykinin, angiotensin 1,Glu^1^- fibrinopeptide B and neurotensin were used in their lyophilized form. In particular, a stock solution was prepared by adding 100 μL of standard diluent (AB Sciex, 30% acetonitrile in 0.01% TFA) in one vial containing 2.3, 4.2, 5.1 and 0.2 μg of des-Arg^1^-bradykinin, angiotensin 1,Glu^1^- fibrinopeptide B and neurotensin, respectively. The stock solution was then diluted 1:10 in 0.1% TFA. Two microliters were then mixed with 5 μL of 0.1% TFA solution to obtain our standard diluted solution with the following concentrations: 0.726 pmol/µL of des-Arg1-bradykinin (MH + 905.05), 0.926 pmol/µL of angiotensin 1 (MH + 1297.51), 0.929 pmol/µL of Glu1- fibrinopeptide B (MH + 1571.61), and 0.034 pmol/µL of neurotensin (MH + 1673.96). An adequate volume of 0.1% TFA was added to standard solution in order to obtain 1:100, 1:400, 1:800, 1:8000 and 1:12,000 serial dilutions. The concentrations of each peptide standards in the serial diluted solutions are reported in Table 1. For MALDI-TOF MS sample preparation CHCA was prepared (4 mg/mL 50/50 ACN/0.1% TFA), then 1 μL of sample was mixed with four μL of matrix and a total volume of 1 μL of the analyte/matrix mixture was deposited on the stainless steel MALDI target plate. For the preconcentration experiments, 2 mg of HMS particles were dispersed in each of the serial diluted solutions (100 μL) and after ten minutes of incubation, the supernatant was removed by gentle centrifugation (2000× *g*) in a sprout® mini-centrifuge (HEATHROW Scientific® LLC, Vernon Hills, IL, USA). The retained peptides were then eluted from the HMS particles directly with 4 μL of CHCA matrix solution (4 mg/mL 50/50 ACN/0.1% TFA), after shaking and vigorously vortexing the suspension for 30 seconds. After centrifugation at 4000× *g*, the solution was removed from the particles and was spotted on the MALDI target plate (three spots of 1 μL). For comparison, the five serially diluted solutions without enrichment were spotted on the MALDI target plate. Specifically, three spots were deposited on the plate and analysed for one sample each time and repeated at least for three times. All the spots with and without enrichment were detected in the same experimental session by MALDI-TOF MS. MALDI measurements wereperformed on a MALDI-TOF mass spectrometer (Voyager DE-STR Applied Biosystems, Foster City, CA, USA) equipped with a 337 nm nitrogen laser. Mass spectra were acquired in linear mode. External mass calibration was performed before MALDI measurements using peptide standards for MS, prepared from the peptide mass standards kit calibration mixture 1 containing des-Arg1-Bradykinin (MH + 905.05), angiotensin I (MH + 1297.51), Glu^1^- fibrinopeptide B (MH + 1571.61), and neurotensin (MH + 1673.96). The acceleration voltage was 20 kV, the guide wire was 0.05% of the accelerating voltage, the grid voltage was 91.5%, and the delay time was 220 ns. Four 100-laser shots were averaged for each mass spectrum. All spectra were processed using Data Explorer Software version 4.11 (AB SCIEX, Framingham, MA, USA).

### 2.3. Sputum Collection

Expectorate was collected from a male chronic obstructive pulmonary disease (COPD) patient diagnosed according to GOLD guideline 2017. Signed informed consent from the participating subject was obtained. Spontaneously expectorated (sputum) was collected into a universal sterile wide mouthed container with a screw cap after rinsing the mouth twice with water in order to avoid oral contamination. The sputum sample was processed immediately after collection, following the protocol described below. 

### 2.4. Sputum Processing

Sputum was treated with four volumes (*w*/*v*) of PBS, then, DTT was added to obtain a final concentration of 5mM DTT. A protease inhibitor cocktail (PIC) (P8340 Sigma-Aldrich) was added to the sputum (15 µL/g of sputum). The tube containing the sample was placed on a bench roller for 15 min at room temperature and the content was filtered through a 70-µm nylon filter (Sigma-Aldrich, St. Louis, MO, USA) and then centrifuged at 400× *g* for 10 min at 4 °C to separate the cells and debris from the liquid phase. The supernatant was carefully removed and re-centrifuged at 12,000× *g*, for 10 min at 4 °C, before being aliquoted and stored at −80 °C.

### 2.5. Sputum Processing with HMS

The protein content of the fluid phase of the sputum was determined by a Bradford assay (1.30 mg/mL). A total of 23 μL were concentrated by vacuum centrifugation (Concentrator 5301, Eppendorf, Hauppauge, New York, USA) to 15 μL, in order to obtain a final concentration of 2 μg/μL and a total protein content of 30 μg. Aliquots (2 mg) of HMS were mixed with 30 μL of diluted sputum fluid phase sample (15 μL of sputum in 15 μL of deionized water) and shaken at room temperature for 15 minutes. The suspension was centrifuged at 2000× g for 2 min, then HMS were separated from the supernatant and washed twice with 0.1% TFA (20 μL). After the last wash, species retained on HMS were extracted with 20 μL of a 1:1 (*v*/*v*) solution of ACN/0.1% TFA and 1 μL of this solution was mixed with 4 μL of 4mg/mL CHCA solution (50/50 ACN/0.1% TFA) for MALDI-TOF MS analysis.

### 2.6. SF Collection and Treatment with Hyaluronidase. 

Osteoarthritis staging and SF sample collection from a grade 2 osteoarthritis patient during total knee arthroplasty were previously described [24]. After collection SF was processed as previously described [25], aliquoted and immediately stored at −80 °C, until used. Hyaluronidase (HSE) (Sigma-Aldrich, St. Louis, MO, USA) stock solution (1300 units/mL) was prepared in SHSE buffer (60 mM NaOAc, 1 mM EDTA, pH 6.0). A total of 500 μL of SF were then mixed with 20 μL of SHSE buffer solution (1.5 M NaOAc, 25 mM EDTA) and with 100 μL of the HSE stock solution. The resulting mixture, was incubated at 37 °C for one hour, subsequently centrifuged at 15,000× *g* for 15 min to precipitate insoluble material. Once separated from the pellet, the supernatant was subjected to pre-analytical treatments and then to MS analysis.

### 2.7. Ultrafiltration Procedure for SF and HSE-treated SF

In order to enrich the low-molecular-weight SF proteome an ultrafiltration step was performed. A total of 500 μL of SF or HSE-treated SF were diluted in 2300 μL of 50 mM ammonium bicarbonate solution. 500 μL of the resulting mixture were placed in a centrifugal filter device (Amicon Ultra 0.5 mL centrifugal filters, Merck KGaA, Darmstadt, Germany), with a nominal molecular mass limit of 3 kDa, and centrifuged at 10,000× *g* for 50 min at 4 °C. In order to extract remaining peptides, the membrane was subsequently washed with 150 μL of 50 mM ammonium bicarbonate solution and centrifuged again at 10,000 g for 10 min at 4 °C. 10 μL of the resulting filtrate were further concentrated by means of SPE with ZipTip C18 pipette tips (Merck KGaA, Darmstadt, Germany) according to the protocol provided by the manufacturer. The peptides bound to the resin were directly eluted using 2.5 μL of a matrix solution, made up of 4 mg/mL CHCA in a 70/30 (*v*/*v*) mixture of ACN and 0.1% TFA. 1 μL of this eluate was then deposited on MALDI target plate and analysed by MALDI-TOF MS.

### 2.8. SF Processing with HMS

A total of 2 mg of HMS were dispersed in 120 μL of diluted SF sample (20 μL in 100 μL of 50 mM ammonium bicarbonate pH 7.8) and shaken at room temperature for 15 min. The dispersion was centrifuged at 600× *g* for 2 min, and then HMS microparticles were separated from the supernatant and rapidly washed twice with 0.1% TFA (20 μL). After the last wash, species retained on HMS were extracted in 30 μL of eluting solution (75:25 ACN/0.1% TFA). The eluate was immediately prepared for MALDI-TOF MS analysis.

### 2.9. Sputum and SF MALDI-TOF MS 

MALDI MS analysis was performed on a MALDI-TOF MS (Voyager DE-STR, Applied Biosystems, Foster City, CA, USA) equipped with a 337-nm nitrogen laser. External mass calibration was performed using calibration mixture 2 prepared from the peptide mass standards kit (AB Sciex, Framingham, MA, USA) containing angiotensin I (MH + 1297.51), ACTH (clip 1–17) (MH + 2094.46), ACTH (clip 18-39) (MH + 2466.72), ACTH (clip 7-38) (MH + 3660.19) and insulin (bovine) (MH + 5734.59) for linear mode. Calibration mixture 1, containing des-Arg1-bradykinin (MH + 905.05), Angiotensin I (MH + 1297.51), Glu^1^- fibrinopeptide B (MH + 1571.61), and neurotensin (MH + 1673.96), was used for spectra acquisition in reflector mode. Spectra acquisition was performed both in linear and in reflectron positive ion mode and delayed extraction was applied. In linear mode, the following settings were used: acceleration voltage 20 kV, guide wire 0.05% of the accelerating voltage, grid voltage 91.5% and delay time 220 ns. In reflectron mode, the following settings were used: acceleration voltage 20 kV, grid voltage 68.5%, mirror voltage ratio 1.12, extraction delay time 300 ns, low mass gate 600 Da. For each spectrum, four 100-laser shots were averaged. Data Explorer Software (version 4.11, AB SCIEX, Framingham, MA, USA).) was used to process all spectra.

### 2.10. Reproducibility

To assess method reproducibility, the same sample was processed in three independent experiments; for each experiment, the sample was run in triplicate, thus, for each preparation, three spectra were acquired, resulting in a total of nine MALDI mass spectra. Spectra acquired were processed by Data Explorer Software. Briefly, each spectrum was normalized, baseline corrected and a smoothing was applied. Peak lists containing peak areas, peak heights, and S/N were exported to Microsoft Excel (Microsoft Corporation®, Redmond, WA, USA) from Data Explorer Software. Twenty selected peaks for each spectrum were compared between runs for, peak height, peak area, and S/N. The mean percentage coefficient of variation (CV%) was calculated from the ratio between SD and the mean of peak height, peak area and S/N.

## 3. Results and Discussion 

### 3.1. Preconcentration of Extremely Diluted Solutions of Standard Peptides

A strategy to decrease the limit of detection of extremely diluted analytes is to concentrate them, as it happens in the case of ELISA, in which the analytes are concentrated by mean of antigen-antibody interaction. Therefore, we tested whether HMS are able to concentrate peptides present at extremely low concentrations.

In order to study the performances and the usefulness of the HMS for MALDI-TOF based-clinical peptidomics, we performed exploratory experiments on the pre-concentration efficiency of these mesoporous silicas. Their enrichment capacity was assessed by using a mixture of four standard peptides in serial dilutions experiments. Therefore, solutions of four standard peptides were prepared from low sub-nanomolar to nanomolar range (Table 1) and processed using HMS according to the extraction protocol, as described in the materials and methods section.

In line with our previous experiments aimed at obtaining a smart and easy protocol for fast and convenient MALDI-TOF profiling procedure [12,13,16,17,24,26,27,28], we performed a dispersive SPE. 

**Figure 1:**
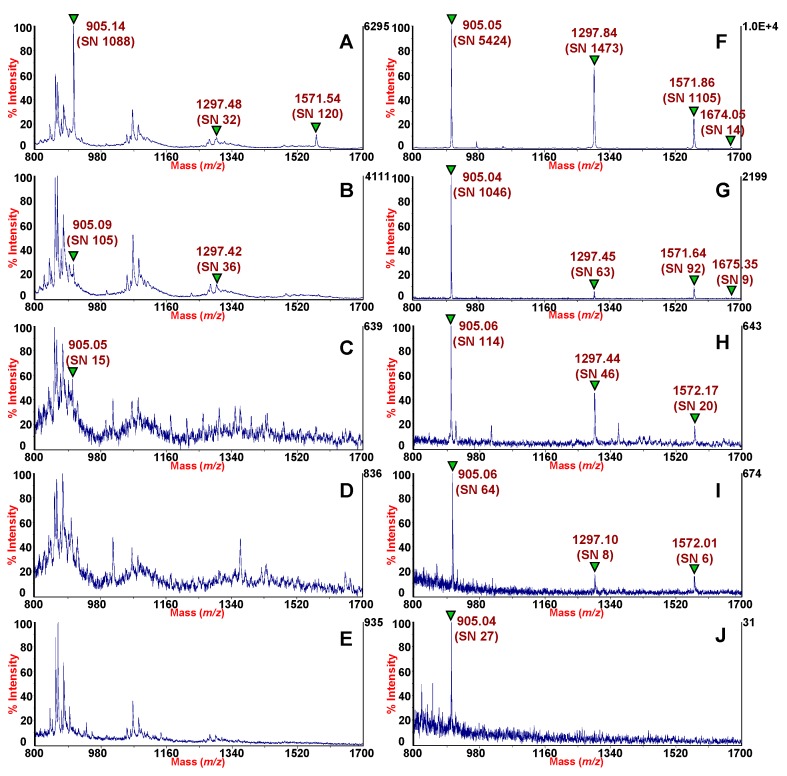
MALDI-TOF MS spectra of the mixture of four peptides: des-Arg1-bradykinin, angiotensin I, and [glu1]-fibrinopeptide B and neurotensin. The serially diluted solutions (left side) (**A**)–(**E**) are compared with MALDI-TOF MS spectra of the same solutions pre-concentrated with HMS (right side) (**F**)–(**J**). Amounts in fmol of each peptide from the serially diluted solutions deposited on the MALDI target plate in a volume of 1 μL are (**A**): 1.45 fmol des-Arg^1^-bradykinin; 1.85 fmol angiotensin I; 1.86 fmol [Glu^1^]-fibrinopeptide B; 0.07 fmol neurotensin; (**B**): 0.36 fmol des-Arg^1^-bradykinin; 0.46 fmol angiotensin I; 0.47 fmol [Glu^1^]-fibrinopeptide B; 0.02 fmol neurotensin; (**C**): 0.18 fmol des-Arg^1^-bradykinin; 0.23 fmol angiotensin I; 0.23 fmol [Glu^1^]-fibrinopeptide B; 0.01 fmol neurotensin; (**D**): 0.02 fmol des-Arg^1^-bradykinin; 0.02 fmol angiotensin I; 0.02 fmol [Glu^1^]-fibrinopeptide B; 0.001 fmol neurotensin; (**E**): 0.01 fmol des-Arg^1^-bradykinin; 0.02 fmol angiotensin I; 0.02 fmol [Glu^1^]-fibrinopeptide B; 0.001 fmol neurotensin. The signal-to-noise ratios are shown in the brackets for each detected peak. All spectra were acquired in linear mode in the same experimental session.

The HMS particles were dispersed into the solution of four standard peptides and after ten minutes of incubation the supernatant was removed by centrifugation, then the CHCA matrix solution was used to directly elute the peptides from the HMS particles, as described in Materials and Methods. The HMS silica were separated from the elution solution and then one μL of this eluate was deposited on the target plate for the MALDI-TOF MS analysis. Both diluted and pre-concentrated solutions were analyzed by MALDI-TOF MS and compared. The gain in (signal-to-noise ratio) S/N of the peaks corresponding to standard peptides in pre-concentrated solution was used as a measure of the HMS efficiency. Figure 1 shows MALDI-TOF mass spectra of the four peptides des-Arg^1^-bradykinin, angiotensin I, and [Glu^1^]-fibrinopeptide B and neurotensin, obtained without any pretreatment (A, B, C, D, E) in serial dilutions (panels on the left), and pre-concentrated by using HMS silicas (F, G, H, I, J) (panels on the right). In panel B and C the concentrations of each peptide is four- and eight-fold lower, respectively, in comparison to panel A, while in panel D and E the concentration of each peptide is 80- and 120-fold lower, respectively in comparison to panel A. 

The typical background interference observed in the MALDI-TOF mass spectra of serially diluted solutions (Figure 1A–E), depends on matrix-cluster formation. In this case (in the serially diluted solutions) the peak of des-Arg^1^-bradykinin (*m/z* = 905) was detected with a high S/N = 1088 only in the MALDI-TOF spectrum acquired for solution A (Figure 1 A). In the other cases, it was difficult to discern this peak from the spectral patterns of matrix adducts (Figure 1B–E). Very low intensity signals were detected for the peaks corresponding to angiotensin I (*m/z* = 1297) and [Glu^1^]-fibrinopeptide B (*m/z* = 1571), which completely disappears from the spectra at the lowest concentrations (Figure 1D,E). The peak corresponding to neurotensin (*m/z* = 1674) was not detected in all of the diluted solutions due to its extremely low concentration (the lowest of the four standard peptides tested). On the right side of Figure 1, the results of enrichment obtained by dispersive SPE using HMS silica are shown. From the comparison of the side-by-side MALDI-TOF mass spectra, the two most evident differences are the dramatic decrease of background originating by the matrix clusters and the gain in the S/N of the pre-concentrated peptides, reported in parentheses in Figure 1. Among all pre-concentrated peptides, the peak of des-Arg^1^-Bradykinin was detected in all of the MALDI-TOF spectra as shown in Figure 1F–J). S/N differences between pre-concentrated and diluted standard indicated gains of 27–4000 units for des-Arg^1^-bradykinin peaks, which was indicative of the enhancement in sensitivity obtained with the pre-concentration procedure. We were able to detect a MALDI-TOF signal of des-Arg^1^-bradykinin with concentrations as low as 0.060 nM equivalent to 0.055 pg/µL (Table 1, Figure 1E,J). In the case of the other peptides, detection limits were 0.116 nM for both angiotensin (Table 1, Figure 1D,I) and Glu^1^- fibrinopeptide B (Table 1, Figure 1D,I), and 0.085 nM (0.14 pg/µL) for neurotensin (Table 1, Figure 1B,G). These results can be considered highly satisfactory in term of sensitivity thus demonstrating that HMS ensures exceptional adsorption capacity with detection limits as low as 0.06–0.12 fmol/µL depending on the standard peptides assayed. Generally, the adsorption process of biomolecules into porous material is controlled by a variety of factors. Among those, the most accountable are hydrophobic and electrostatic interactions [29]. Other influencing factors may include the experimental conditions such as temperature, pH of solutions, the isoelectric point of the analytes, the pore size and symmetry [13]. HMS is characterized by small domain size with large textural mesoporosity and short channels [30]. These peculiar features of HMS might provide more suited transport channels for peptides to access the internal surface of the materials. Furthermore, we argue that the outstanding adsorption capacity observed can be ascribed also to the wormhole like mesoscopic assembly of the HMS.

### 3.2. Analysis of Peptidic Profiles of Clinical Samples Sputum and SF

**Figure 2:**
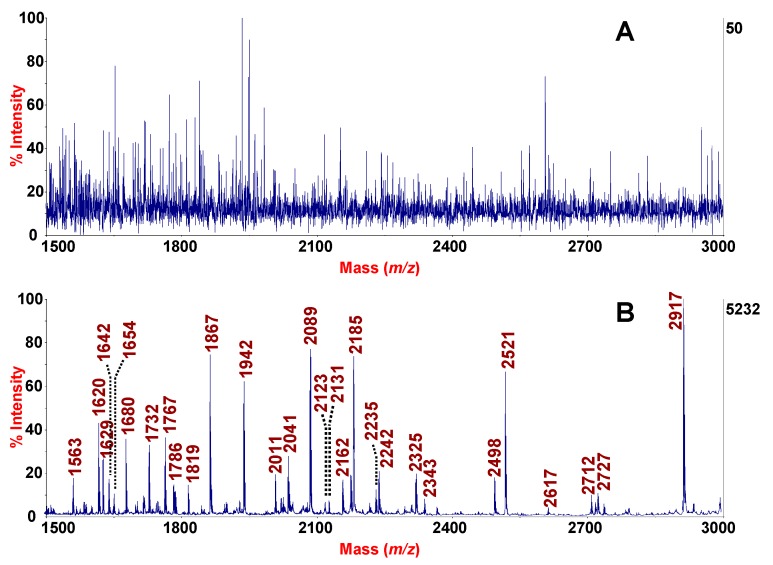
MALDI-TOF MS profiles, obtained after processing sputum from a patient suffering of COPD without enrichment (**A**) and with enrichment by dispersive solid phase extraction by HMS (**B**). The spectra were acquired in reflector mode in the range 700–5000. Monoisotopic peaks are labelled in the *m/z* range 1500–3000.

**Figure 3:**
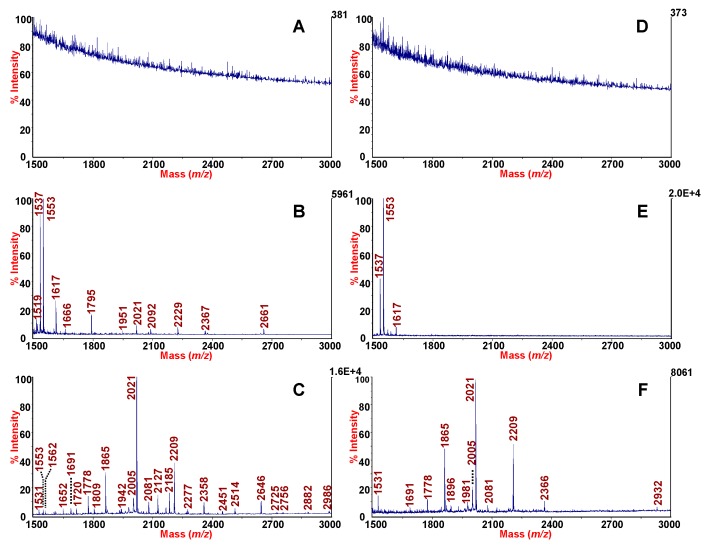
MALDI-TOF MS profiles of SF (**A**), SF after ultrafiltration with Amicon centrifugal filter devices followed by ZipTip C18 (**B**) and SF after enrichment by using HMS microparticles (**C**). MALDI-TOF MS spectra of HSE-treated SF (**D**), HSE-treated SF after ultrafiltration with Amicon centrifugal filter devices followed by ZipTip C18 (**E**) and HSE-treated SF after enrichment by using HMS microparticles (**F**). The spectra were acquired in reflector mode in the range 700–5000. Monoisotopic peaks are labelled in the range from 1500 to 3000.

MALDI-TOF MS-based profiling strategies of biofluids present a direct avenue for biomarker discovery and clinical practice [6,31]. Although MALDI-TOF is highly sensitivity and tolerant of salts and buffers, direct peptidomics profiling of several bodily fluids is often unsuccessful. Particularly, the presence of large and abundant proteins confoundsthe detection of peptides. An example is shown by the spectrum of the fluid phase of expectorate sputum sample in Figure 2A. The spectrum indeed, shows high noising background with marked signal suppression. Similarly, MALDI-TOF mass spectra with absence of signals are acquired also in the case of intact SF (Figure 3A). Treatment of clinical specimen for peptide extraction and sample preparation is therefore of crucial importance.

The use of mesostructured HMS whormole silicas was, therefore, tested on sputum and SF in order to harvest the peptidic components excluding from the adsorptive process large size proteins and polymers, which hindered the detection of peptides.

Sputum from a patient suffering from COPD and SF from a subject diagnosed with arthritis were used in order to demonstrate the applicability of the HMS to harvest and enrich these clinical specimens in their peptidic components.

### 3.3. HMS-MALDI-TOF MS Sputum Profile

In the case of sputum, we analyzed the spontaneous expectorate from a patient suffering from COPD, a lung disease which is the fourth leading cause of death worldwide, which is characterized by symptoms such as dyspnea, wheezing, cough, and, among other symptoms also by sputum production [32]. More precisely, spontaneous sputum production occurs in a subset of patients suffering from COPD. This fluid is very important from a clinical point of view because it provides information about both inflammatory cells and mediators present in the airways, which accurately mirror inflammatory changes at the site of tissue damage [33]. Since COPD lacks established reliable biomarkers to be used for prognosis and to assess treatment effectiveness, sputum could be an important source of potential laboratory markers for COPD [26].

Sputum is a complex secretion and one of major hurdle encountered in peptidomics investigations is the presence of large, heavily glycosylated and highly charged mucins, which interfere with detection of peptides in MS analyses. Moreover, the use of DTT to reduce disulphide bridges in sputum is not enough to overcome this problem. In fact, as shown in Figure 2A, the MALDI-TOF MS spectrum of the fluid phase of sputum despite use of the reducing agent DTT, is dominated only by background noise with absence of signals. 

In order to extrapolate peptidic signatures associated to COPD, we used a rapid procedure based on the use of HMS as “molecular sieves” to separate high molecular weight sputum proteins from low molecular weight sputum peptides. In line with our previous protocol for induced sputum [26], based on the use of MSB, an analogous workflow was adopted. Analytical conditions, specifically, adsorption times, amount of HMS-to-sputum volume, elution volumes were tested and varied, with the aim to the enhance extraction efficiency and the sensitivity of the analysis which was measured as function of the number of the peaks detected by MALDI-TOF MS (data not shown). The protocol performed with HMS allows to obtain spectra with intense peaks especially in the range between 1500 and 3000 and to analyse minor peptidic components with a high resolution (Figure 2B). The number of peaks detected as function of S/N ratio is reported in Table 2. In particular, the number of peaks detected in the range from 700–5000 with a S/N greater than 30 was 498 which increased to 741 for a S/N ≥ 20 (Table 2). The reproducibility of the protocol developed was evaluated by technical replicates as described in Materials and methods section and the results are summarized in Table S1 and Figure S1. In the sputum experiments, mean percentage CVs ranged from 0.34–17.96 for peak heights, from 0.62–17.28 for peak areas and from 0.34 to 17.69 for the S/N, thus ensuring a good reproducibility for MALDI MS-based profiling studies. 

### 3.4. Enrichment of Naturally Occurring Peptides from SF

SF is a highly viscous fluid located in the joints with lubricating and nutritive functions. SF contains a large number of proteins originating from serum and synovial surrounding tissue [34] and also glycoproteins and carbohydrate polymers of hyaluronic acid. SF is considered a rich source of biomarkers for pathologies such as rheumatoid arthritis and osteoarthritis because it is in direct contact with the affected tissues, cartilage, synovial membrane and bone [35]. However, the presence of large size and high abundance (1–3 mg/mL) of hyaluronans (large carbohydrate polymers of hyaluronic acid) seriously hinders the MALDI-MS detection of peptides in unprocessed sample of SF. In order to facilitate both sample pre-treatment and MS analysis, SF is frequently enzymatically digested with HSE so that its viscosity is reduced by breaking the chains of hyaluronic acid [36]. This pre-treatment is then followed by an ultrafiltration step with commercially available centrifugal filter device with a nominal molecular mass limit in a range from 3–10 kDa [37]. A final step for further concentrating and desalting by means of SPE with ZipTip C18 pipette tips is necessary before MALDI-TOF MS analysis [7]. Therefore, this conventional approach is very expensive not only for the high purchase costs of the filter/SPE devices but also because it requires very long processing time (see Materials and Method, Section 2.7).

We have previously demonstrated that, when SF was treated with mesoporous aluminosilicate (MPAS), due to a cut-off mechanism, hyaluronans are excluded while peptides are captured [24]. The protocol proposed with MPAS was practical, efficient and rapid with the acquisition of rich peptide profiles of SF without the use of HSE. In line with the previous study, in the present investigation we assessed the performance of mesostructured HMS for selective binding and enrichment of SF peptidome repertoire. The textural properties of the HMS silica are quite similar to those of MPAS in terms of pore size, high surface area and high pore volume (Table 3). However, differently from MPAS, the HMS mesostructures appear as spongelike particles with the presence of a characteristic wormhole framework domain [38]. 

We treated SF with or without HSE, before HMS-MALDI-TOF MS analysis in order to compare the better strategy in term of peak yield. The results obtained when using HMS for SF are illustrated in Figure 3C,F. Figure 3C,F show the peptidome fingerprintings obtained with our procedure from the SF of a patient suffering from osteoarthritis after HMS treatment without and with the use of HSE respectively. Compared to the controls (see Figure 3A,D), the use of HMS significantly amplified the peptides repertoire of SF as expected. The best performance was obtained when using HMS procedure preceded by HSE digestion, which resulted in the detection of 681 peaks as compared to the 582 detected without HSE treatment (Table 4). However, in both cases the use of HMS resulted very satisfactory by increasing the peptide yield in comparison to control sample. The comparison in peak number between MPAS and HMS (Table 4) shows that a better performance was obtained when using HMS. Compared to MPAS, the smaller particle size of these sorbents, and the presence of wormhole framework domains, makes more accessible the entrance in the pores and the entrapping of the analytes [21,22]. 

A good reproducibility was warranted by this procedure for both SF and HSE-SF. Three representative mass spectra replicates are shown in Figure S2. As reported in Table S1, in the case of SF mean percentage CVs ranged from 0.51–14.95 for peak heights, from 1.47–14.92 for peak areas and from 0.52–17.07 for the S/N. Similar ranges of variations were also observed in the case of HSE-SF. 

The use of commercially-available Amicon Ultra 0.5 mL centrifugal filter devices with a molecular cut-off of 3000 Da followed by Zip Tip C18 sample preparation was also assessed for both intact (Figure 3B) and HSE-treated SF (Figure 3E). The number of peaks detected in the *m/z* range from 700 to 5000 with S/N > 30 was higher when HSE was used (Table 4). In the case of centrifugal filter devices/Zip Tip procedure, the average number of peaks detected was 345 for SF treated with HSE in comparison to the 303 peaks detected without HSE treatment. The use of HMS was preferable to the Amicon centrifugal filter devices followed by ZipTip C18. A significant increase in the number of detected peaks was obtained when the HMS were used in comparison to centrifugal filter/Zip Tip procedure.

Although in the case of filtration device followed by ZipTip the number of peaks detected were also satisfactory as shown in Table 4 (303 and 345), however, a more extensive and time-expensive two-steps pre-treatment was required before MALDI-TOF MS analysis.

As it happened in the case of the MPAS, the high capacity recovery, (expressed as function of MALDI-TOF number of peaks), was also observed for HMS silicas. It is worth noting that, as for MPAS, in this case the procedure was also based on a dispersive SPE, in which a slurry is prepared by suspending the sorbent phase in the sample solution [12]. In this extraction procedure, the contact area between the solid phase and the analytes is extremely maximised over the conventional SPE thus allowing a better and more effective interaction, thus reducing the time needed for performing the protocol (only 15 minutes for the adsorption times). Other advantages of the procedure described are the low amounts of silica sorbents used (only two mg for both SF and sputum) and the low volumes of solvents with an improved analytical performance, which can also be assayed by the high resolution of the peptide peak detected.

**Figure 4:**
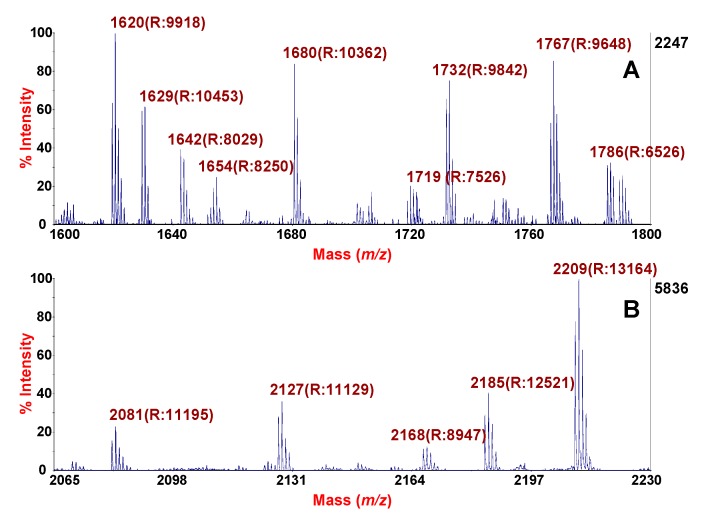
MALDI-TOF spectra of sputum (**A**) and SF (**B**) after HMS treatment. Specific *m/z* ranges with labeled monoisotopic peaks. For each peak, the resolution is indicated in parentheses. The spectra were acquired in reflector mode.

MALDI-TOF MS spectra zoomed in *m/z* ranges 1600–1800, and 2065–2230 are shown in Figure 4 to better highlight the excellent resolution of the analysis, both for sputum (Figure 4A) and for SF (Figure 4B). These specific *m/z* ranges are reported in order to highlight, for each spectrum, the high resolution obtained for peaks of low, medium and high intensity. For each peak, the resolution is shown in parentheses. The spectra were acquired in reflector mode. The quality of mass profile is an important pre-requisite to ensure comparative analyses as well as peptide/protein identification in clinical biomarker discovery. Specifically, in top-down experiments, due to the difficulties encountered in sequencing endogenous peptides, high intensity/resolved precursor peaks are required for accurate MS/MS identification.

## 4. Conclusions

Reducing the high complexity of clinical samples for improving the detection of peptidome signature in MALDI-TOF biomarker discovery is a very challenging task. We have described the use of HMS for pre-concentrating, desalting and peptide enrichment from both diluted and complex mixtures, such as SF and sputum. The protocol based on a dispersive-SPE is rapid, allowing an improved analytical performance with high quality MALDI-TOF MS spectra.

The data show that the pre-concentration processes based on the use of HMS constitutes a good approach for the detection of low molecular weight peptides present in a very diluted sample. The textural properties of these silica sorbents coupled to the characteristic wormhole framework were satisfactory for integrating size selectivity with efficient adsorptive mechanism. Highly-enriched and well-resolved MALDI-TOF peptidic profiles were acquired for complex clinical samples of sputum and SF. These exploratory experiments demonstrate that HMS are very suitable for harvesting rich peptide fractions of SF and sputum, by a selective, sized-controlled and effective host-guest interaction.

Currently, assays based on a clinical MS proteomics and/or peptidomics strategy, owing to their requirements for skilled operators, are more complex to implement than conventional assays. Moreover, they have not been validated at the level needed for clinical applications. In fact, the biomarkers analysis assays presently used in medical laboratories meet both laboratory and clinical requirements and have been already optimized in term of turn-around-time, ease and analytical performances [39]. Therefore, assays based on a clinical MS proteomics and/or peptidomics strategy need to become more robust, faster and user friendly. The protocol proposed as “dispersive” SPE is rapid, economical and ensures good analytical performances. The possibility of developing smart and versatile protocols, allowing selective isolation of peptides prior to MS analysis, as described in this study, may accelerate the expected translation of clinical peptidomics into medical practice.

## Figures and Tables

**Table 1:** Concentrations of standard peptides in the serially diluted solutions.

Standard Peptides (*m/z*)	Serial Dilutions				
	1:100	1:400	1:800	1:8000	1:12000
des-Arg^1^-Bradykinin (905)	7.26 nM (6.571 pg/µL)	1.82 nM (1.642 pg/µL)	0.90 nM (0.821 pg/µL)	0.090 nM (0.082 pg/µL)	0.060 nM (0.055 pg/µL)
Angiotensin I (1297)	9.26 nM (12.015 pg/µL)	2.32 nM (3.004 pg/µL)	1.16 nM (1.502 pg/µL)	0.116 nM (0.150 pg/µL)	0.078 nM (0.100 pg/µL)
Glu^1^- Fibrinopeptide B (1571)	9.29 nM (14.6 pg/µL)	2.32 nM (3.65 pg/µL)	1.16 nM (1.825 pg/µL)	0.116 nM (0.183 pg/µL)	0.078 nM (0.122 pg/µL)
Neurotensin (1674)	0.34 nM (0.569 pg/µL)	0.085 nM (0.140 pg/µL)	0.043 nM (0.071 pg/µL)	0.004 nM (0.007 pg/µL)	0.003 nM (0.005 pg/µL)

**Table 2:** Number of peaks detected in MALDI-TOF MS spectra with S/N ≥ 20 and S/N ≥ 0 from sputum.

Sample	Bead Type	Average ^a)^ Number of Peaks S/N > 20	Average ^a)^ Number of Peaks S/N > 30
Sputum	N/A ^b)^	25 ± 2	13 ± 2
Sputum	HMS	741 ± 27	498 ± 35

a) Average number of peaks detected is calculated on three replicate spectra acquired in reflector mode in an *m/z* range from 700 to 5000 in CHCA matrix solution; b) HMS processing not applied.

**Table 3:** Textural properties of HMS and MPAS.

MPS	Pore Size Diameter (nm)	Pore Volume (cm^3^/g)	BET Surface Area (m^2^/g)
HMS	2–4	1–2	800–1000
MPAS	3.0	0.90	937

**Table 4:** Number of peaks detected in MALDI-TOF MS spectra with S/N ≥ 20 and S/N ≥ 30 from intact or HSE-treated SF samples processed with and without HMS, MPAS and Amicon centrifugal filter-C18.

Sample	Bead Type	Average ^a)^ Number of Peaks S/N > 20	Average ^a)^ Number of Peaks S/N > 30
SF	N/A ^)^	0 ± 0	0 ± 0
SF	Amicon Centrifugal filter-C18	480 ± 31	303 ± 34
SF	HMS	765 ± 15	582 ± 11
HSE-SF	N/A^b)^	12 ± 1	2 ± 1
HSE-SF	Amicon Centrifugal filter-C18	488 ± 9	345 ± 11
HSE-SF	HMS	967 ± 39	681 ± 6
SF	MPAS	668 ± 84	367 ± 55

a) Average number of peaks is calculated on three replicate spectra acquired in reflector mode in an *m/z* range from 700 to 5000 in CHCA matrix solution; b) HMS processing Not Applied.

## Supplementary Materials

Supplementary materials are available online. Figure S1. Reproducibility studies on sputum peptidome profiling; Figure S2. Reproducibility studies on SF peptidome profiling; Table S1. Reproducibility assessment for peak height, peak area and S/N in acquired MALDI-TOF mass spectra from three independent experiments with HMS.
