# Supplementary material for: Hexagonal Mesoporous Silica as a Rapid, Efficient and Versatile Tool for MALDI-TOF MS Sample Preparation in Clinical Peptidomics Analysis: A Pilot Study
Source: Molecules. 2019 Jun 22;24(12):2311. doi: 10.3390/molecules24122311 (PMC6631377; doi:10.3390/molecules24122311)
Supplement: Supplementary file 1 [file molecules-24-02311-s001.pdf]

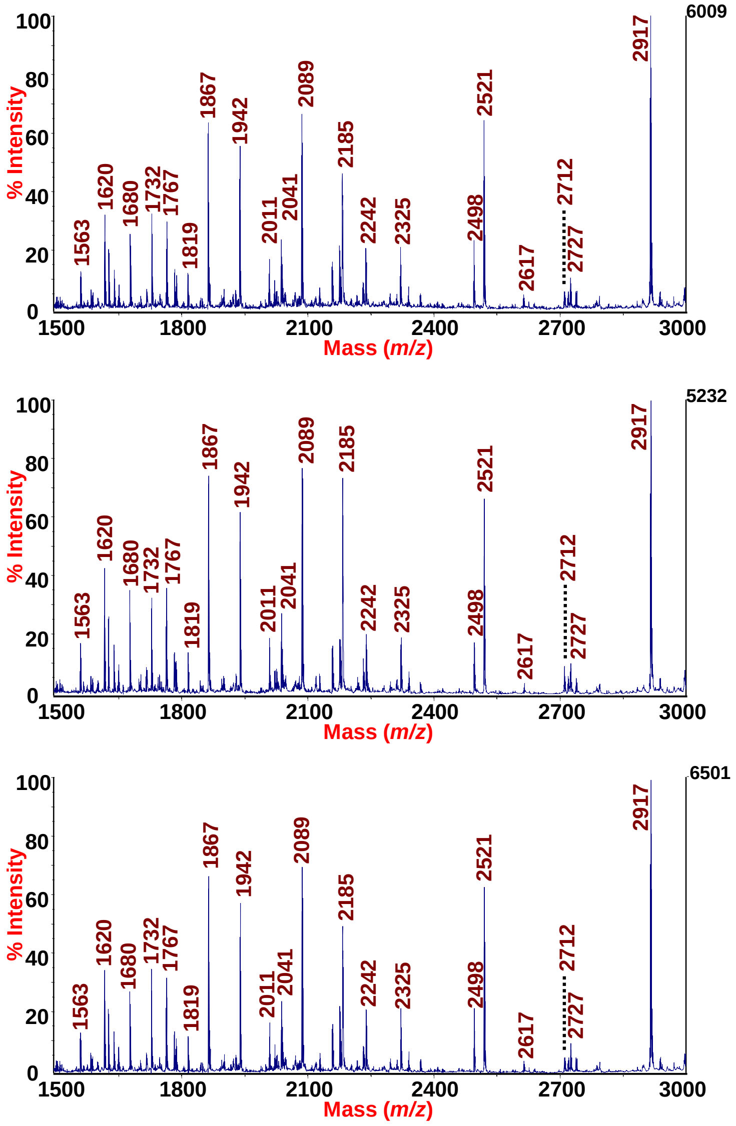

Figure 1. Reproducibility studies on sputum peptidome profiling. MALDI-TOF mass spectra replicates of sputum were obtained from the same patient after HMS enrichment. The spectra were acquired in reflector mode using CHCA matrix.

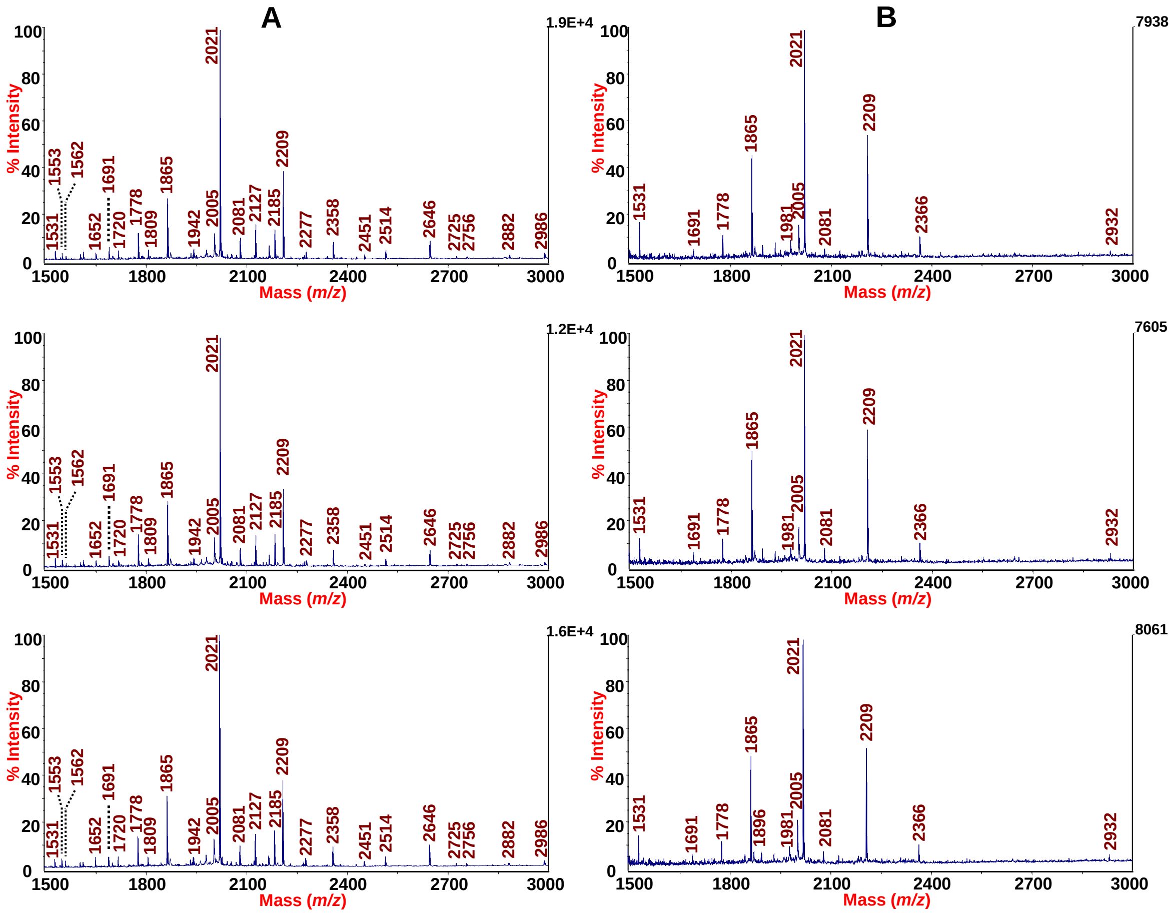

Figure 2. Reproducibility studies on SF peptidome profiling. MALDI-TOF mass spectra replicates of SF were obtained from the same patient after HMS treatment without (column A) and with (column B) the use of HSE. The spectra were acquired in reflector mode using CHCA matrix.

Table 1. Reproducibility assessment for peak height, peak area and S/N in acquired MALDI-TOF mass spectra from three independent experiments with HMS.

Sputum-HMS				SF-HMS				HSE-SF-HMS			
m/z	CV (%)[a)] height	CV (%)[a)] area	CV (%)[a)] S/N	*m/z*	CV (%)[a)] height	CV (%)[a)] area	CV (%)[a)] S/N	*m/z*	CV (%)[a)] height	CV (%)[a)] area	CV (%)[a)] S/N
**1287**	4.34	4.62	7.12	**905**	6.08	11.98	9.05	**905**	3.61	13.98	3.82
**1335**	1.42	3.69	8.66	**1450**	10.73	7.61	1.22	**1061**	13.89	11.72	17.44
**1434**	10.80	4.21	3.98	**1531**	10.07	10.26	8.89	**1077**	11.43	8.28	19.21
**1563**	15.60	15.45	16.47	**1553**	2.86	11.86	13.00	**1212**	15.24	9.57	3.81
**1620**	10.26	17.28	0.34	**1562**	14.84	11.87	17.07	**1329**	16.52	8.72	22.59
**1680**	1.82	7.40	13.74	**1652**	9.71	10.57	11.62	**1349**	14.96	8.61	13.43
**1732**	17.96	15.48	14.79	**1691**	10.31	8.50	1.52	**1450**	11.95	4.81	9.34
**1767**	12.18	6.96	1.19	**1720**	14.03	7.80	14.75	**1499**	18.72	8.22	23.59
**1867**	6.02	1.12	9.84	**1778**	5.15	14.11	5.15	**1531**	15.01	11.26	12.96
**1942**	7.42	2.22	1.50	**1865**	5.07	6.43	6.65	**1691**	14.25	12.55	1.08
**2041**	1.94	2.26	3.30	**1942**	0.51	3.40	2.26	**1778**	14.20	5.94	0.95
**2089**	6.85	3.19	4.01	**2005**	9.19	4.95	6.28	**1865**	1.63	4.79	7.02
**2185**	14.24	3.18	17.69	**2021**	14.95	4.32	13.05	**1896**	8.61	5.51	16.64
**2325**	0.34	0.62	12.08	**2081**	9.23	14.29	0.52	**1935**	16.47	20.17	17.31
**2343**	4.63	3.17	5.87	**2127**	12.88	12.12	10.10	**1981**	13.80	5.62	14.13
**2498**	1.70	0.68	9.07	**2185**	12.49	4.75	15.25	**2005**	4.87	1.82	14.63
**2521**	6.11	6.42	2.37	**2209**	12.74	1.47	14.87	**2021**	6.73	5.27	6.34
**2917**	4.26	4.22	1.11	**2358**	10.73	11.64	14.23	**2081**	9.47	8.03	6.15
**3474**	10.18	14.24	10.68	**2514**	6.30	14.92	8.67	**2209**	2.94	9.08	10.19
**4369**	3.36	8.80	6.62	**2646**	8.51	13.62	8.45	**2365**	3.54	17.51	11.51

a) Mean percentage coefficient of variation (CV%) calculated from 20 peaks in three independent experiments with HMS. For each experiment the sample was run in triplicate; consequently for each preparation three spectra were acquired resulting in a total of nine MALDI mass spectra. Spectra were acquired in reflector mode in an *m/z* range from 700–5000 using CHCA matrix solution.
